# BMI Course Over 10 Years After Bariatric Surgery and Biopsychosocial Complexity Assessed with the INTERMED: a Retrospective Study

**DOI:** 10.1007/s11695-021-05440-8

**Published:** 2021-05-12

**Authors:** Yann Corminboeuf, Beate Wild, Catherine Zdrojewski, Dieter Schellberg, Lucie Favre, Michel Suter, Friedrich Stiefel

**Affiliations:** 1 Psychiatric Liaison Service, Lausanne University Hospital and University of Lausanne, Lausanne, Switzerland; 2 Department of General Internal Medicine and Psychosomatics, Medical University Hospital, Heidelberg, Germany; 3 Service of Endocrinology, Diabetes and Metabolism, Lausanne University Hospital and University of Lausanne, Lausanne, Switzerland; 4 Department of Surgery, Riviera-Chablais Hospital, Aigle-Monthey, Switzerland

**Keywords:** Obesity, Bariatric surgery, INTERMED, Case complexity, Outcome

## Abstract

**Background:**

While bariatric surgery is an effective therapy for patients with severe obesity, not all patients benefit equally. An explanation might be that psychosocial risk factors hamper outcome. The study aimed to evaluate if biopsychosocial case complexity predicts evolution of BMI over 10 years after bariatric surgery.

**Methods:**

Charts of patients (*N* = 236) of the Cohort of Obesity Lausanne (COOL) were retrospectively reviewed and rated with the INTERMED, a reliable and validated instrument, which assesses biopsychosocial case complexity and has been proven to predict outcome of medical treatments in different patient populations. The sample was stratified into BMI quartiles, computed from the patients’ baseline BMI. For each quartile, BMI evolution was analyzed using individual growth curve analysis.

**Results:**

Growth curve analyses showed that in quartiles 1, 2, and 3, none of the INTERMED domain scores significantly predicted the BMI evolution after surgery. However, in the fourth quartile—including patients with the highest pre-surgical BMI—the social domain score of the INTERMED significantly predicted BMI evolution: patients with more social complexity showed higher increase in BMI.

**Conclusion:**

Effectiveness of interventions targeted at social complexity, especially when patients suffer from severe obesity, may therefore be evaluated in future studies.

**Graphical abstract:**

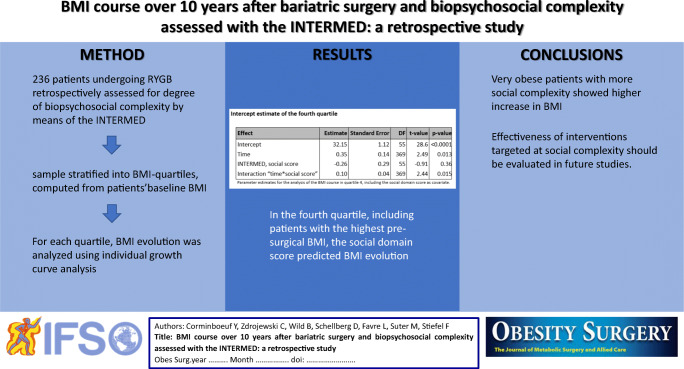

## Introduction

Obesity, defined as excessive fat accumulation that may impair health, concerns 650 million of adult aged 18 years and over [1]. Obesity has nearly tripled in many European countries since the 1980s [2], and its progression continues. Treatments for obesity include lifestyle interventions, pharmacotherapy, and bariatric surgery [3]. Many individuals struggle to maintain weight loss with lifestyle interventions, and although the landscape of obesity pharmacotherapies is rapidly changing, the long-term results of treatments like GLP-1 analogs has yet to be demonstrated [4, 5]. On the other hand, bariatric surgery achieves sustained reduction in weight, improvement of co-morbidities, and prolonged survival [6]. The Roux-en-Y gastric bypass (RYGBP) and the sleeve gastrectomy are currently the most commonly performed operations worldwide and considered by many clinicians and researchers as gold standard. Cohorts—such as the Cohort of Obesity Lausanne (COOL)—have thus been created to investigate long-term effects of surgery [7]. Even though the majority of patients seem to benefit from bariatric surgery, not all benefit equally. However, it remains impossible to identify patients who will show insufficient weight loss or important weight regain after surgery [8]; this subgroup with a diminished response—defined as less than 20% of weight loss—concerns 19 to 35% of patients 10 years after surgery (RYGBP) [9–11].

Both, biological and psychosocial factors might explain diminished treatment response in patients who undergo obesity surgery. However, evidence-based psychosocial outcome prediction of obese patients who undergo surgery is lacking [11, 12].

The aim of the present study was to evaluate if the INTERMED—a biopsychosocial instrument to assess case complexity and to identify patients with poor response to medical treatments [13]—also predicts less favorable outcome in the long term after RYGB.

## Methods

### Study Design and Participants

Participants were recruited from the COOL, which was initiated in March 2015 (including a retrospective arm since 1999) with the aim to investigate long-term evolution after bariatric surgery and to determine prognostic factors [14]. Patients (*N* = 272) were randomly selected from the COOL (*N* = 1600), and their charts assessed by means of the INTERMED. All patients underwent RYGBP.

Retrospective studies with the INTERMED based on chart review have already been conducted [15, 16]. Since patients included in the COOL undergo a comprehensively documented medical and psychiatric assessment, the two investigators (CZ and YC) who filled in the INTERMED did not encounter any particular problems. However, for some patients (*N* = 36), the social domain score of the INTERMED could not be rated based on the information available in the charts; these patients were excluded from the study.

### INTERMED: Assessment of Biopsychosocial Case Complexity

Medical complexity is defined as coexisting conditions, which hamper the success of medical care [17]. Beside disease- and treatment-related parameters, psychosocial factors and aspects situated at the interface between the patient and the health care system, increase medical complexity. The INTERMED (see Table 1), an instrument to assess biopsychosocial case complexity, has been demonstrated to identify patients with a diminished response to medical treatments [18–20] in many different patient populations and clinical settings, such as low back pain [21, 22], chronic shoulder pain [23], diabetes [24], palliative care [25], or internal medicine [26]. Research has also shown that early psychosocial interventions targeted at complex patients identified by means of the INTERMED improve their outcome [27, 28].

**Table 1:** The INTERMED

	History	Current state	Prognosis
**Biological**	Chronicity Diagnostic uncertainty	Severity of illness Diagnostic uncertainty	Complications and life threat
**Psychological**	Restrictions in coping	Resistance to treatment	Mental health threat
	Premorbid psychiatric dysfunctioning	Severity of psychiatric symptoms	
**Social**	Restrictions in social integration	Residential instability	Social vulnerability
	Social dysfunctioning	Restrictions of social network	
**Health care**	Intensity of prior treatment	Organizational complexity	Care needs
	Prior treatment experience	Appropriateness	

Source: Huyse et al. [18]

The INTERMED integrates information from four domains—biological, psychological, social, and healthcare related—assessed in a temporal perspective—history, current state, and prognosis. The eight domains referring to the past (history) and present (current state) contain 2 items, and the four referring to the future (prognosis) contain 1 item. Each item is scored between 0 and 3, depending on the degree it contributes to case complexity (higher scores indicating a higher case complexity). Given that there are 20 items, case complexity can range from 0 (absence of case complexity) to 60 (highest biopsychosocial case complexity) [19]. Based on pooled outcomes of INTERMED studies, a cut-off score of 21, which divides patients in non-complex and complex patients, has been identified [29].

The INTERMED can be used as an observer-rated instrument based on an interview or filled in retrospectively based on chart reviews. An INTERMED self-assessment, which has been shown to correlate with the observer-rated version, is also available [30].

### Interrater Reliability Testing with the INTERMED

At the beginning of the study the first 46 charts were double scored by the two trained INTERMED raters and interrater reliability (ICC) was calculated. Since the ICC for the INTERMED sum score was 0.91 indicating a high interrater reliability, subsequent charts were scored by only one rater (YC).

### Statistical Analyses

Initially, descriptive statistics for BMI and INTERMED scores were calculated and presented (mean value ± standard deviation (SD)).

The main analysis was carried out using an individual growth model (Singer et al, 1998).

For the analysis, we first examined the distribution of pre-surgical BMI values of the study sample (because baseline BMI determines the BMI course after surgery). With a minimum baseline BMI of 34.0 and a maximum of 73.4 kg/m^2^, the study sample showed a high heterogeneity regarding pre-surgical BMI. We therefore stratified the sample into BMI quartiles computed from the individual’s baseline BMI. Stratum 1 included the 25% of the sample within the lowest pre-surgical BMI quartile, and stratum 4, respectively, the 25% within the highest pre-surgical BMI quartile.

The prediction of the BMI courses was then modelled for each quartile separately. For each stratum, we ran four regression models (resulting in 16 different regression analyses) to investigate whether the variation in intercepts and slopes of the individual’s BMI curves was related to an INTERMED domain score (biological, psychological, social, or health care use).

**Fig. 1:**
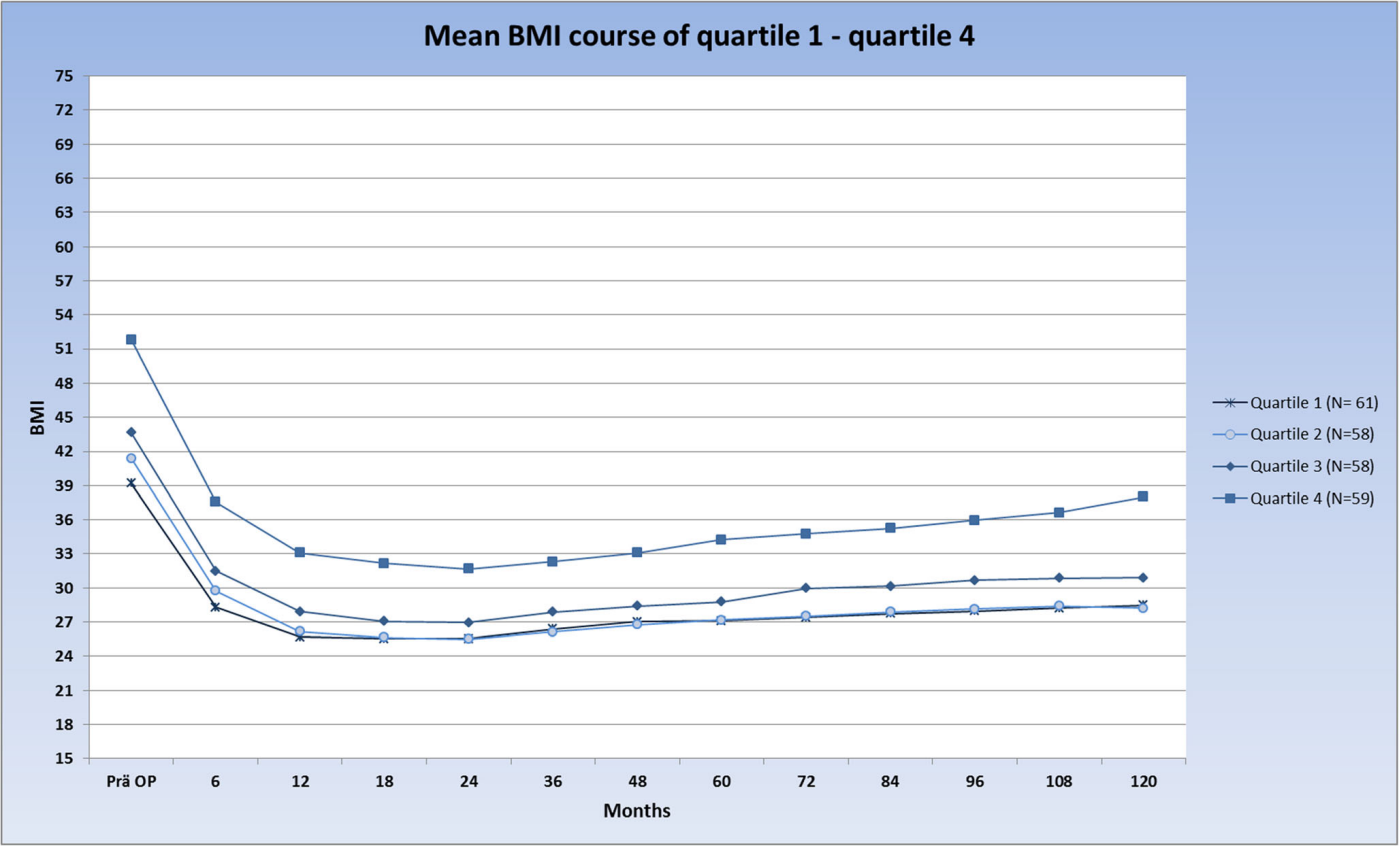
BMI course of the patients

For the modelling of the growth curves, we had to consider that the mean BMI course of the patients is strongly decreasing until 2 years after surgery (Fig. 1). Afterwards, the BMI course slowly starts to increase. We therefore included in the growth curve analyses the BMI values of 2–10 years after surgery.

The conditional growth curve models were fitted as linear growth models with “time” as fixed effect. The BMI values measured at 2–10 years after surgery were entered as repeated measurements. In the regression model, an INTERMED domain score was included as covariate—as well as its interaction with time. The parameter estimate for the interaction of an INTERMED domain score with time indicates the differences in growth rates with respect to the domain score. For a significant time × domain score interaction, we finally examined the explained variation attributable to the domain score by comparing the estimates from the unconditional model (without covariate) with the estimates from the conditional growth model. All analyses were conducted by using SAS, version 9.4 (SAS Institute Inc., 2017).

## Results

The baseline characteristics of the selected patients are shown in Table 2. No significant difference was observed between selected (*N* = 236) and excluded patients (*N* = 36).

**Table 2:** Baseline characteristics of patients

	Included patients (*n* = 236)		Excluded patients (*n* = 36)	
	*N*	%	*n*	%
Gender				
Female	197	83.5	30	83.3
Male	39	16.5	6	16.7
	**Mean value (SD)**	**[Min;Max]**	**Mean value (SD)**	**[Min;Max]**
Age (years)	38.1 (9.5)	[20;61]	37.5 (10.3)	[19:65]
Pre-OP BMI	44.0 (5.8)	[34;73.4]	45.1 (5.2)	[39.5:59.2]
BMI (24 months post-OP)	27.4 (4.9)	[18.7;50.9]	28.3 (5.2)	[21.9;40.5]
BMI (120 months post-OP) (*n* = 137)	31.8 (7.3)	[18.1;63.1]	29.9 (4.4)	[22.8;39.2]
INTERMED biological domain	8.7 (1.8)	[5;12]	7.7 (1.7)	[5;10]
INTERMED psychological domain	5.7 (2.7)	[1;12]	5.0 (2.6)	[3;8]
INTERMED social domain	2.9 (3.9)	[0;13]	–	–
INTERMED health care use	3.1 (1.7)	[1;8]	2.8 (0.8)	[2;4]
INTERMED total scores	20.4 (7.3)	[8;40]	–	–

Demographic characteristics of study participants and persons excluded due to missing values

Mean pre-surgical (baseline) BMI of included patients (*N* = 236) was 44.0 ± 5.8 with BMIs ranging from 34.0 to 73.4.

The mean INTERMED total score (20.4 ± 7.3) shows a significant overall heterogeneity, with scores ranging from 8 to 40, and high biopsychosocial complexity of the sample. The biological domain (8.7 ± 1.8) shows the highest score, reflecting the serious health concerns of these patients. The psychological domain (5.7 ± 2.7) also shows high scores and psychological heterogeneity.

**Fig. 2:**
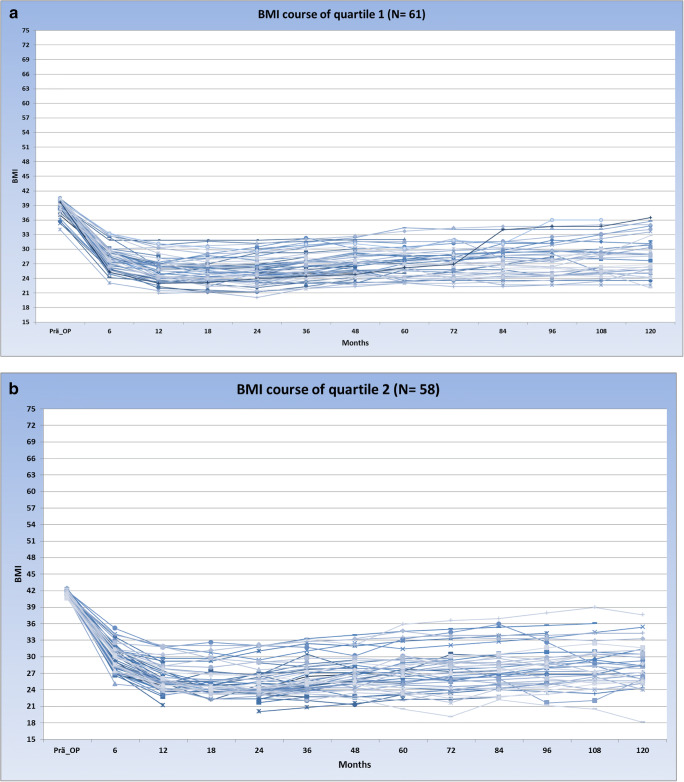

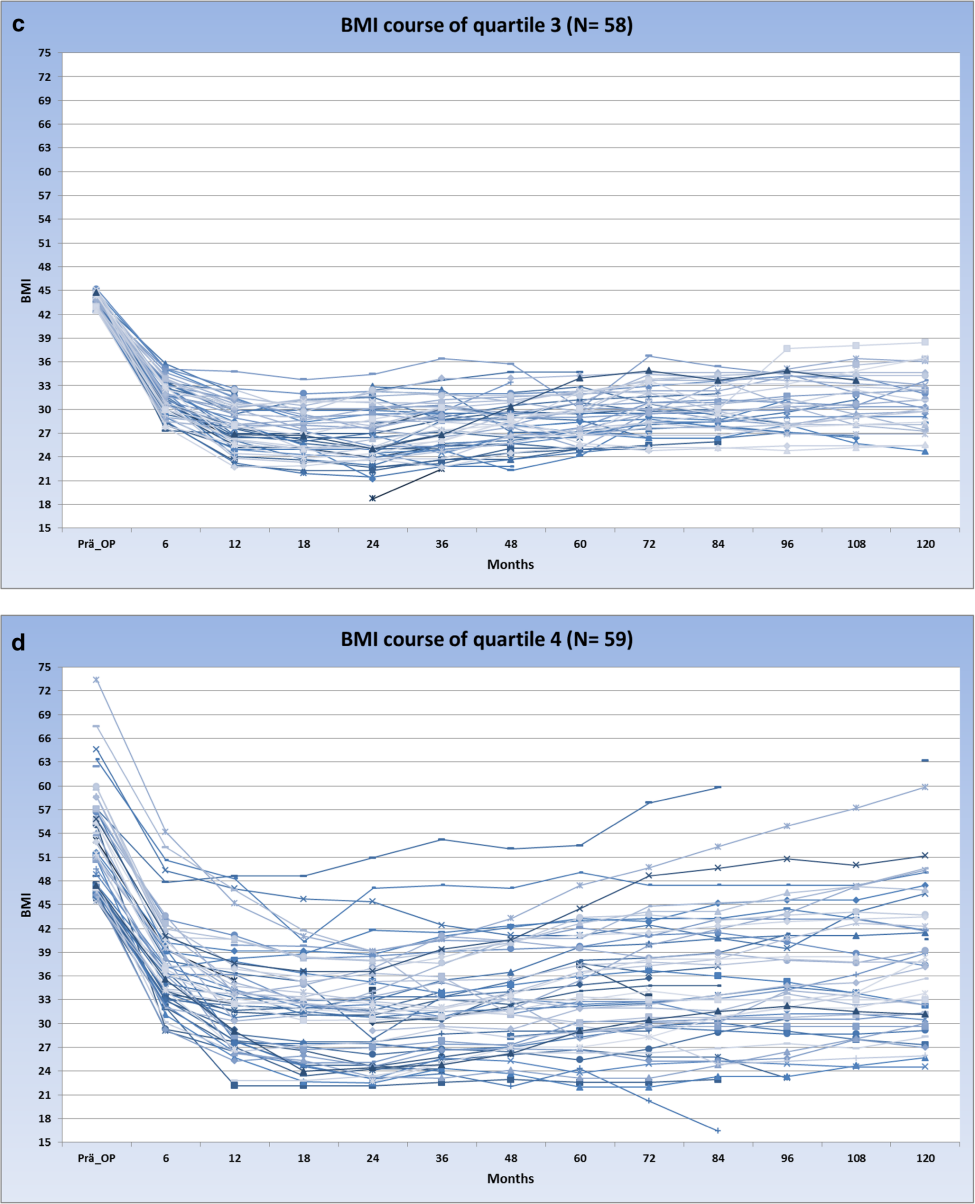
**a**–**d** Courses of the patients over 10 years after bariatric surgery are shown

Based on the high variability in pre-surgical BMI, the study sample was divided into quartiles. The mean pre-surgical BMI was 39.2 ± 1.4 in the first quartile, 41.4 ± 0.6 in the second, 43.9 ± 0.9 in the third, and 51.9 ± 5.9 in the fourth quartile. The biopsychosocial complexity in the four quartiles was quite similar with mean values of total INTERMED scores ranging between 19.7 (± 7.1, quartile 3)) and 21.2 (±7.1) (quartile 4). In Fig. 2a–d, the courses of the patients over 10 years after bariatric surgery are shown, stratified into four groups according to their baseline BMI.

**Fig. 3:**
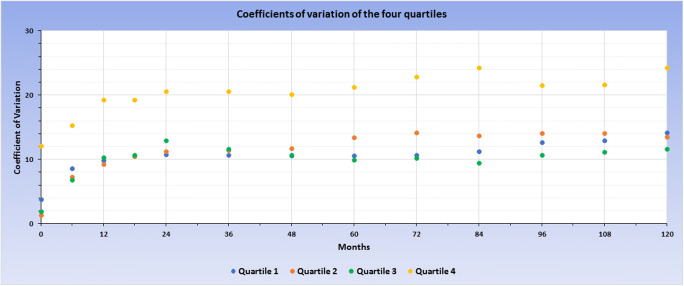
CV of quartiles 1 to 4

The courses of the strata indicate that the group with the lowest baseline BMIs exhibits a relatively homogenous BMI course over the 10 years after surgery. However, the standard deviation of BMI courses is increasing over the four groups. In the fourth stratum with the highest pre-surgical BMI, the BMI courses after surgery are highly heterogeneous. To quantify the degree of variation within the four different strata the coefficients of variation (CVs)—defined as the ratio of the respective standard deviation to the mean value—were calculated for each time point. Figure 3 depicts that at all measurement points, the CV of the fourth quartile was substantially higher compared to the CVs of quartiles 1–3, reflecting the greater variability of BMI values in quartile 4.

Growth curve analyses showed that in quartiles 1, 2, and 3, none of the INTERMED domain scores is a significant predictor of the BMI course after surgery. However, in the fourth quartile, the social domain score of the INTERMED significantly predicts the course of the 10-year BMI course.

The intercept estimate shows that the average BMI of the fourth quartile was 32.1 at 2 years after surgery (see Table 3). The time estimate reflects the average slope of the BMI course. Hence, the average patient of the fourth quartile began with a BMI of 32.1 ± 1.12 at 2 years after surgery and gained 0.35 BMI points each year thereafter. The estimate for the covariate “social domain score” indicates that there is no significant relationship between BMI at 2 years after surgery and the social INTERMED domain (*p* = 0.36). However, the significant parameter estimates of 0.10 for the interaction indicates that persons with a higher score in the INTERMED social domain have a higher slope in the BMI curve over 10 years after surgery (*p* = 0.015). A comparison between the unconditional model and the model including the social domain score as covariate shows that the social domain score explains 9% of the variance in growth rates.

**Table 3:** Intercept estimate of the fourth quartile

Effect	Estimate	Standard error	*df*	*t* value	*p* value
Intercept	32.15	1.12	55	28.6	<0.0001
Time	0.35	0.14	369	2.49	0.013
INTERMED, social score	−0.26	0.29	55	−0.91	0.36
Interaction “time * social score”	0.10	0.04	369	2.44	0.015

Parameter estimates for the analysis of the BMI course in quartile 4, including the social domain score as covariate

## Discussion

This is the first study which uses the INTERMED in a population of patients with obesity. The percentage of patients in this sample (42.3%) showing complexity (INTERMED score ≥21) is about one-third higher compared to other samples of patients with chronic diseases treated in a tertiary care center [24, 27]. Psychological and social problems in chronic diseases, such as diabetes, rheumatoid arthritis, or low back pain may be pre-existent or independent of the diseases, a consequence of the disease or simply lacking. In severe obesity, the psychological, social, and somatic aspects are often interwoven since psychosocial distress may provoke obesity and obesity may provoke psychosocial stress through stigmatization.

Despite the variability of biopsychosocial complexity, INTERMED scores did not predict courses of BMI after surgery. This result may indicate that the high percentage of complex patients diminished the predictive power of the INTERMED. However, it may also be, that “weight loss after bariatric surgery” is determined by various and yet unknown factors and thus not comparable with outcomes such as HbA1c levels [31] or hypertension and hyperlipidemia [32].

Almost all patients show rapid weight loss in the first 2 years after bariatric surgery. The INTERMED scores, if assessed for example after the second year, may also have changed compared with baseline, and thus gained in predictive power. What is needed, however, is an outcome prediction instrument that can be used prior to surgery. Moreover, all except the quartile with the highest baseline BMIs showed low outcome variation, which may also explain the limits of the INTERMED regarding outcome prediction. In the quartile with the highest baseline BMI and the highest outcome variation, mean biopsychosocial complexity measured with the INTERMED was not different from the other quartiles, but the social domain of the INTERMED significantly predicted BMI courses over time and explained 9% of the variance in growth rates. How can we understand these last results?

It is interesting that heterogeneity regarding surgery outcomes in obese patients increases, depending on BMI at baseline. This may be due to different reasons. First, the increased possible amount of total weight loss in patients who have very high BMI scores increases the possibility of variance with regard to outcomes [33]. Second, more severe eating disorder, associated with psychiatric comorbidities, may hamper favorable long-term outcome and favor weight regain [34–37]. Third, surgery can have both negative and positive effects, which is difficult to predict, as it also requires psychological adaptation to a modified body.

While these reasons may explain outcome variability, the fact remains that 9% of the variance in growth rates can be explained by the social domain of the INTERMED at baseline. The higher the social problems at baseline, the more patients from the fourth quartile increase in BMI in the course of 2–10 years after surgery. The explanation is likely to be multifactorial. Patients suffering from severe obesity, more stigmatized by the social environment, have more difficulties to remain mobile and socially active and more often lose their work [38]. In addition, considering the important psychosocial stress and high psychiatric morbidity associated with obesity [39], the sole buffer for coping with obesity might be social support. Social support not only beneficially influence psychiatric morbidity and eating disorders after bariatric surgery [40] but is also a decisive factor for greater weight loss after surgery, and social support plays an important role with regard to treatment adherence [41] and motivation for change [42]. Social problems are also associated with chronic stress and higher cortisol levels, which can promote long-term weight regain [43]; moreover, patients with low socio-economic status are physically less active [44].

Finally, the observation that the mean social domain score in the quartile with the highest BMI baseline scores was similar to the mean social domain score in the quartile with the lowest BMI baseline score is a last interesting result. We hypothesize that the social domain score only plays a role in groups with higher outcome heterogeneity, thanks to the buffering effect of “the social” (social support, social network, social isolation). Alfa Wali et al. have studied the impact of socio-economic deprivation on bariatric weight loss outcomes and demonstrated that there was no significant difference in outcomes, according to deprivation level up to 2 years after surgical intervention, which is in line with our results for this follow-up time [45].

To summarize: the INTERMED failed to predict weight loss after RYGB, for the reasons mentioned above, but this study reveals a clinically relevant and sometimes neglected aspect of obesity care, the social difficulties associated with obesity. While psychiatric evaluation and treatment is an integral part of obesity care, a systematic assessment of the social situation by social workers is lacking in many centers, as it is the case in ours. At least in our center, social workers are only included in the treatment, when the endocrinologist, surgeon or psychologist/psychiatrist refer the patient. Our study indicates, however, that availability and easy access to social assessment, counselling, and help should probably be part of obesity treatment since it might improve results.

Our study has methodological strengths, such as the long-term follow-up and having used all the available BMI measurements over time (by modelling growth rates). This allowed to observe that BMI courses differ widely depending on pre-surgical BMI and that social factors might play a decisive role in the very obese with the highest outcome variance.

A limitation of the study is its retrospective character and its sample size. However, retrospective studies based on chart review have already been conducted with the INTERMED and interrater-reliability of the INTERMED assessments was very high. Given the sample size, a replication of the prognostic value of the INTERMED social score in a larger sample would be useful.

## Conclusion

Our study showed that outcome in terms of weight loss among patients with more-severe obesity (quartile 4) undergoing RYGB differ more in long-term BMI course compared with patients suffering from less-severe obesity (quartiles 1, 2, and 3). Despite a wide heterogeneity of the quartile 4 population, the social domain of the INTERMED explained 9% of variability of the long-term post-operative weight at 10 years. Interventions aiming the assessment of, and support in the social dimensions for patients with very severe obesity, should therefore be evaluated in future studies, since they might influence weight outcome. Further studies will have to prove if early social interventions, complementing psychiatric care for those who are in need, will be beneficial.
